# PlmCas12e Utilizes Glu662 to Prevent Cleavage Site Occupation by Positively Charged Residues Before Target Strand Cleavage

**DOI:** 10.3390/molecules29215036

**Published:** 2024-10-25

**Authors:** Jinchu Liu, Lizhe Zhu

**Affiliations:** School of Medicine, and Warshel Institute for Computational Biology, The Chinese University of Hong Kong (Shenzhen), Shenzhen 518172, China; 222050017@link.cuhk.edu.cn

**Keywords:** CRISPR-Cas12e, CasX, gene editing, molecular dynamics, TAPS algorithm, path searching, minimum free energy path, enhanced sampling

## Abstract

CRISPR-Cas12e is a recently identified gene-editing tool mainly known because its relatively small size benefits cell delivery. Drastically different from Cas9, it creates a blunt-end double-strand breakage of the DNA via two cleavage sites; Cas12e produces a sticky-end double-strand breakage of the DNA through only one cleavage site in its RuvC domain, meaning two consecutive cleavage events first on the non-target strand (ntsDNA) and then the target strand (tsDNA). Though crucial for Cas12e’s cleavage efficiency, the mechanism by which Cas12e loads tsDNA for the second cleavage remains elusive. Through molecular dynamics simulations and our recently matured traveling-salesman-based automated path-searching (TAPS) algorithm, we identified a series of positively charged residues (Arg856^TSL^, Arg768^RuvC^, Lys898^TSL^, Arg904^TSL^, Arg764^RuvC^) that guide the tsDNA backbone toward the cleavage site of wild-type *Plm*Cas12e. Further simulations of the R856L and R904L mutants supported such observations. More interestingly, we found the key role of Glu662^RuvC^ in coordinating Arg764^RuvC^, preventing its occupation of the cleavage site, and facilitating tsDNA cleavage. Additional simulations confirmed that mutating Glu662^RuvC^ to valine disabled such coordination and created a stable intermediate state with Arg764^RuvC^ occupying the cleavage site before tsDNA loading. These insights, revealing an elaborate mechanism of cleavage facilitation, offer essential guiding principles for future rational engineering of Cas12e into more efficient gene-editing tools.

## 1. Introduction

Clustered regularly interspaced short palindromic repeats (CRISPR) and CRISPR-associated (Cas) systems offer adaptive immunity against foreign nucleic acids in bacteria and archaea [1,2,3,4]. The CRISPR-Cas immune defense operates through three critical stages: (a) foreign DNA fragments are integrated into the CRISPR locus, (b) CRISPR RNA (crRNA) is expressed, and (c) the system interferes with the target [5,6]. In the interference stage, crRNA pairs with Cas proteins to create a ribonucleoprotein (RNP) complex that guides the Cas nuclease to its matching DNA target for precise cleavage. This RNA-guided DNA targeting capability of CRISPR-Cas systems has revolutionized genome engineering tools [7,8,9,10]. These systems are divided into two distinct classes based on their effector protein composition [11,12]: Class I systems require multiple Cas proteins for interference, whereas Class II systems need only a single multi-domain Cas protein. Notably, Cas9 and Cas12a, both Class II effectors, have become indispensable in genome editing across various cell types and organisms [13,14,15,16].

Cas12e (also known as CasX) is part of the Class II CRISPR systems. It targets a 5′ thymine-rich protospacer-adjacent motif (PAM) and has shown high genome editing efficiency in human cells when paired with single-guide RNA (sgRNA), combining CRISPR RNA (crRNA) and trans-acting crRNA (tracrRNA) [8,17,18,19,20]. With a compact size of ~980 amino acids, ~30% smaller than *Spy*Cas9 or *As*Cas12a, Cas12e is more suitable for AAV vector delivery for gene therapy [21,22,23]. This makes Cas12e a promising alternative for genome editing alongside Cas9 and Cas12a.

**Figure 1:**
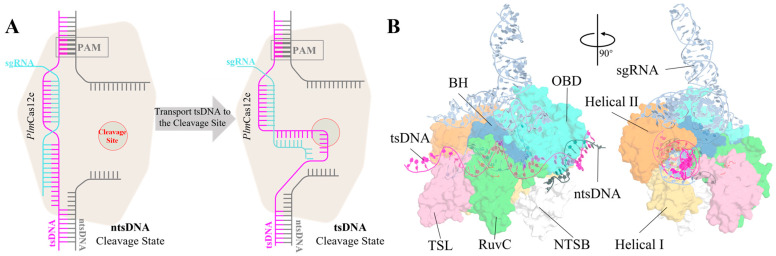
The transition processes underwent molecular dynamics simulation, and the structure of the ternary complex for the *Plm*Cas12e at the ntsDNA cleavage state. (**A**) The biological processes we studied in this paper are how the tsDNA is loaded into the cleavage site. (**B**) The ternary structure of the *Plm*Cas12e in its ntsDNA cleavage state: sgRNA is colored in pale blue, BH is colored in blue, RuvC is colored in green, Helical-II is colored in orange, OBD is colored in cyan, tsDNA is colored in magenta, ntsDNA is colored in grey, Helical-I is colored in yellow, TSL is colored in pink, and NTSB is colored in white.

Cas12e exhibits minimal protein sequence similarity with Cas12a or Cas9, aside from a shared RuvC nuclease domain [18] (Figure 1). Cryo-electron microscopy (cryo-EM) studies of Cas12e–sgRNA–DNA complexes revealed that Cas12e uses its RuvC domain to cleave both the target strand (tsDNA) and non-target strand (ntsDNA) DNA, a mechanism like that of Cas12a [24,25].

Two unique domains in Cas12e are essential for DNA cleavage: the target-strand loading (TSL) domain, positioned near the tsDNA, and the non-target strand binding (NTSB) domain, located next to the ntsDNA. The NTSB domain plays a crucial role in DNA unwinding, as its name suggests, and was proven by experiment as well [8,18]. These structural differences indicate that Cas12e operates through distinct mechanisms compared to other Cas proteins.

Another unique feature of the Cas12e compared to Cas9 is its ability to produce staggered double-strand breaks (DSBs) [8,18,26,27], which could make homology-repair or genomic editing more accurately than the blunt breaks introduced by Cas9 [28,29,30]. These staggered DSBs originate from the only RuvC nuclease domain available in Cas12e: (a) the ntsDNA first disassociates from tsDNA and is cleaved by the RuvC domain; and (b) the tsDNA is then loaded into the cleavage site and cut at a different position, creating staggered DSBs [8,18,19]. Though the first step has been studied recently [19], the kinetic mechanism by which tsDNA is loaded for cleavage remains elusive.

To date, two homologs of Cas12e are frequently applied for gene editing: Cas12e from *Deltaproteobacteria* (*Dpb*Cas12e) and *Planctomycetes* (*Plm*Cas12e). These proteins share 68% sequence similarity and can use the same sgRNA [8,18]. Structure comparison between the two homologs at the same state exhibits extreme similarities (RMSD of 0.8 Å over all the Cα in the secondary structure). Through careful inspection of the structure of Cas12e (Figure S1), we found there are many positively charged residues outside of the cleavage site, mainly on the RuvC and TSL domains, and the connection between the TSL and the RuvC domain is mostly through unstable loops (Figure S1) [8,18]. Due to its higher activity in vivo and higher resolution of the known structures than *DpbCas12e*, we decided to use *Plm*Cas12e as a reference starting point.

Structural biology techniques such as cyro-EM, capable of revealing some stable states of Cas12e, can hardly offer complete mechanistic insights into this tsDNA loading process (i.e., intermediate states, ISs, and transition states, TSs). Brute-force molecular dynamics simulations can, in principle, complement experimental techniques in offering atomic-level mechanisms but they suffer from high time costs, especially for large complex molecules. This necessitates the applications of path methods, such as transition path sampling (TPS) and its derivatives [31,32,33,34], which directly sample transition paths in the phase space, compute the rates, and identify the most relevant degrees of freedom for a transition. Alternatively, one may use path-searching methods that use biased sampling to find the minimum free energy path (MFEP) close to a guessed path, such as fast tomographic [35,36], path-metadynamics [37,38], and finite temperature string [39,40,41]. However, in practice, path-searching approaches often face the limitation that they rely heavily on a priori selected collective variables (CVs), which are challenging to define, especially for large systems.

Here, we use our recently matured traveling-salesman-based automated path-searching (TAPS) algorithm, which alleviates this issue [42,43,44]. TAPS only requires a distance metric as input, i.e., the set of atoms used to compute the root-mean-square distance (RMSD) between any pair of conformations. Such a feature is particularly useful for macromolecules as large as Cas12e, where the vital atoms underlying the conformational change are unknown a priori. Once we obtain, through TAPS, the MFEP connecting the ntsDNA-cleaved and tsDNA-loaded states of Cas12e, umbrella sampling can be performed to obtain the free energy landscape along the MFEP for discovering the critical ISs and TSs.

From the MFEP located by TAPS, we observed five distinct ISs within the path, indicating a six-step process for tsDNA loading by Cas12e. Notably, the structure right before the tsDNA cleavage revealed how specific arrangements of charged residues work coherently with each other to guide the loading of tsDNA into the cleavage site.

## 2. Results

### 2.1. Overall Features of the Free Energy Landscape for tsDNA Loading

**Figure 2:**
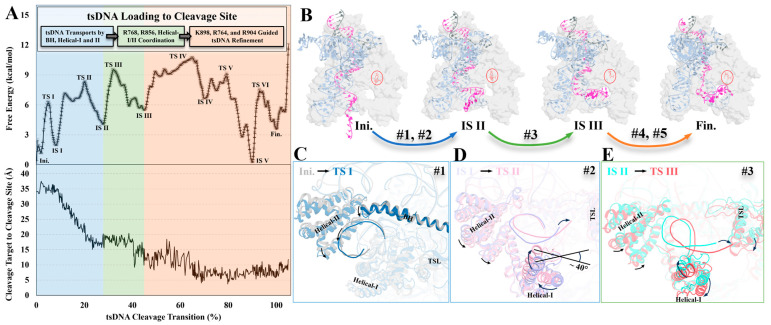
The MFEP of the tsDNA cleavage transition and the significant structural difference during the cleavage transition. Stage 1 is from Ini. to IS II, colored in blue; Stage 2 is from IS II to IS III, colored in green; Stage 3 is from IS III to Fin., colored in orange (**A**). The top one is the MFEP of the transition; the first stage is colored in blue, describing the tsDNA transported by protein domains; the second stage is colored in green, describing residues working in conjunction with protein domains while moving the tsDNA; and the third stage is colored in orange, describing the residues taking control of the tsDNA. The bottom one shows the distance from the tsDNA to the cleavage site during the transition process. (**B**) The structural differences between each IS, the protein is a transparent surface, tsDNA is colored in magenta, sgRNA is colored in pale blue, and ntsDNA is colored in grey; each different colored arrow corresponds to the color of the stage, and red circles are the location of the cleavage site. (**C**–**E**) The enlarged structural changes during the energy barriers.

Our TAPS approach revealed that for the wildtype *Plm*Cas12e enzyme, the transition from the ntsDNA-cleaved to the tsDNA-loaded state is a six-step process comprising five intermediate states (IS) and six transitional states (TS) (Figure S2E). Here, we label the ntsDNA-cleaved state as Ini. on the MFEP, with the tsDNA-loaded state labeled as Fin. We first notice a free energy difference (3.56 kcal/mol) between the Ini. and Fin. states, which is seemingly modest for a multi-domain protein (Figure 2A). Unexpectedly, this transformation from ntsDNA to the tsDNA cleavage state is energetically disfavored. We observe a continuously ascending free energy profile toward IS IV with four forward energy barriers of 6.15, 6.37, 5.47, and 5.24 kcal/mol, respectively. During the transition, the backward barriers are marginally lower, approximately 1–2 kcal/mol less than their forward counterparts (4.28, 4.24, 4.06, and 4.06 kcal/mol) (Figure S2E), which indicates that a reverse process is more likely to occur for IS I, II, and III. Following IS IV, remarkable stability is observed for IS V, which means the complex needs to overcome a substantial energy barrier of 7.16 kcal/mol to reach the tsDNA cleavage state (Figure S2E). Collectively, the endothermic reaction, the backward-favored IS, and the elevated energy barrier at the IS V contribute to a less rapid transition from the Ini. to Fin. states.

A detailed analysis of the trajectory data discloses that non-target strand binding (NTSB) and target strand loading (TSL) exhibit notably higher average root mean square fluctuations (RMSF) in comparison to the OBD, Helical-II, and Bridging-Helix (BH) domains (Figure S2F). As discussed in the introduction, the NTSB domain is bound tightly to the ntsDNA during the disassociation between ntsDNA and plays a lesser role in facilitating the tsDNA loading to the cleavage site. However, the proximity of the TSL domain to the tsDNA, sgRNA, and the RuvC domain strongly indicates its pivotal role in tsDNA loading. In the following two sections, we elaborate on the role of TSL and RuvC.

### 2.2. Stages of tsDNA Loading

For the ease of presenting the detailed mechanism, we categorize the progression from the ntsDNA to the tsDNA cleavage state into three distinctive stages (Figure 2A), each separated by the distance between the cleavage target (tsDNA) and the active cleavage site (Asp659^RuvC^, Glu756^RuvC^, and Asp922^RuvC^).

Stage 1 (blue) is characterized by Helical-I, Helical-II, and BH movements; the cleavage target first oscillates approximately 35 Å away from the cleavage site and then quickly drops off to 17 Å (Figure 2A). This stage is characterized by the presence of two energy barriers (Ini. → TS I and IS I → TS II). Structural assessments during the first barrier (Ini. → TS I) revealed a substantial spatial contraction through bending the BH domain’s tail and the inward movement of Helical I (Figure 2C). These motions shift the R-loop’s backbone formation, resulting in fluctuations in the number of hydrogen bonds between bases within the R-loop, destabilizing the R-loop formation and raising the overall free energy during the process. During the second barrier (IS I → TS II) of Stage 1, the Helical-I domain rotated along its axis (perpendicular to the page) clockwise, together with the inward movement of the Helical-II, bending the already less stable R-loop about 40° (Figure S4A), resulting in the tail of the sgRNA toward the TSL domain by about 4 Å (Figure 2D and Figure S3A). The hydrogen bond number decreases along the motion (Figure S4C).

**Figure 3:**
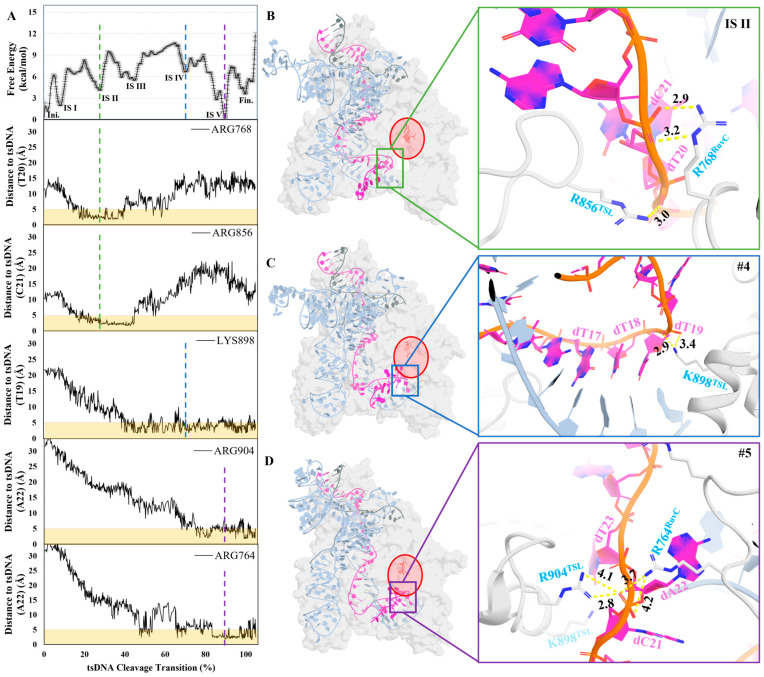
The atomistic structures of the different intermediate states. (**A**) The distance between each of the residues and tsDNA along the MFEP, IS II is in green, IS IV is colored in blue, and IS V is colored in purple; the yellow color indicates the distance threshold of 5 Å. (**B**–**D**) The structures at different intermediate states. The protein is transparent white cartoon or stick mode, sgRNA is colored in pale blue in cartoon mode, tsDNA’s base is colored in magenta, tsDNA’s backbone is colored in orange, and tsDNA is in cartoon or stick mode.

Next is Stage 2 (green), which mainly involves the motions of Helical-I, TSL, and a set of positively charged residues coordinating the DNA backbone. The tail of the sgRNA progressively approaches TSL, facilitated by one more rotation undertaken by the Helical-I domain itself. As in the previous rotation in the first stage, it also resulted in an approximate 4 Å displacement of the tail of the sgRNA toward TSL (Figure 2E and Figure S4B), and this time, the sgRNA captured by TSL further led to a decrease in the accessible volume for tsDNA and therefore its movement (Figure S5). In addition, the positively charged residues Arg768^RuvC^ and Arg856^TSL^ captured the negatively charged backbone of tsDNA like a plier to stabilize or confine tsDNA (Figure 3B).

Stage 3 (orange) can be divided into two significant barriers (IS III → TS IV and IS V → Fin.), both of which witness an expanded control of the tsDNA by the TSL domain. During the first energy barrier (IS III → TS IV), which now seizes a more upstream portion of the sgRNA (Figure S4F), together with a decrease in distance between the TSL and Helical-II domain (Figure S4E), further confines the tsDNA’s movement. As for the second barrier (IS V → Fin.), the significant difference is the orientation of the tsDNA. When crossing the barrier (at TS VI), some bases of the DNA point toward the cleavage site, partially occupying the space for the tsDNA’s backbone and interfering with the cleavage coordination. However, at Fin., the tsDNA’s backbone occupies the cleavage site by itself, and the bases point outwards, no longer interfering with the cleavage site (Figure S3B). During this process, the tsDNA’s backbone moved a controlled amount for refinement; thanks to the positively charged residues such as Arg764^RuvC^, Arg904^TSL^, and Lys955^RuvC^, they prevented the tsDNA backbone from steering away.

### 2.3. Coordination of tsDNA by Positively Charged Residues

We noticed a strong presence of the positively charged residues guiding the orientation of the tsDNA in the previous section (Arg768^RuvC^, Arg856^TSL^, Arg764^RuvC^, Arg904^TSL^, and Lys955^RuvC^). The next question is how the positively charged residues are oriented in the direction to control the tsDNA. During the structural analysis of the MFEP, we found that the positively charged residues are situated on flexible loops, which are meticulously regulated by neighboring negatively charged and polar residues typically embedded within more stable secondary structures such as α-helices or β-sheets (Figure S7). With this observation, we propose that the positively charged residues, instrumental in guiding the tsDNA backbone, are precisely oriented to facilitate the intentional movement of tsDNA during protein compression; while the protein relaxes, these residues ensure that the tsDNA stays in its place without falling back into its location before compression. In fact, during the relaxation of TS III to IS III, the protein’s radius of gyration increased, but the distance between the tsDNA and the cleavage site did not. In other words, it creates a local minimum for the tsDNA to settle in (Figure 3A and Figure S7).

In the early stages of the transition, particularly just before the capture of sgRNA (IS II), Arg768^RuvC^ and Arg856^TSL^ function in tandem to exert precise control over the tsDNA segments encompassing dC21 and dT20. This strategic plier maintains the position of the tsDNA, preventing any backward slippage as Helical-I undergoes its conformational relaxation (Figure 3B and Figure S4D).

Progressing to Stage 3, Lys898^TSL^ engages with the phosphate group of dT19 on the tsDNA, effectively anchoring one end of the DNA, thereby ensuring that the opposing end is free to be captured and stabilized (Figure 3C).

Promptly following Lys898^TSL^’s coordination, in IS V, Arg904^TSL^ and Arg764^RuvC^ collaborate to seize the backbone of dC21, securing the other end of the cleavage target (Figure 3D). This dual capture firmly locks the tsDNA backbone into place, establishing a highly stable structure. With the backbone securely held, the primary remaining task is to orient the nucleotide bases correctly, thereby facilitating the entry of the tsDNA into the cleavage site and efficient cleavage.

**Figure 4:**
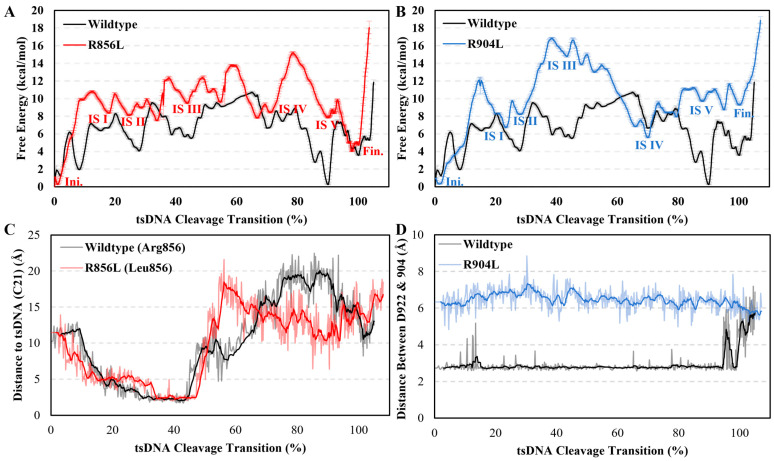
MFEP for the mutations of R856L and R904L. (**A**,**B**) The comparison from R856L and R904L to wildtype MFEP; wildtype in black, R856L colored in red, and R904L colored in blue. (**C**) The distance between the tsDNA and residue 856^TSL^; wildtype colored in black, and the R856L mutation colored in red. (**D**) Distance between the cleavage site and residue 904^TSL^; wildtype colored in black, and the mutation colored in blue.

To validate the hypothesis that positively charged residues are critical during the transition from ntsDNA to tsDNA cleavage, we designed two mutations aimed at partially disrupting this process, namely Arg856 and Arg904 (TSL domain), both to leucine (Leu). Leucine was chosen for substitution to ensure that any observed effects would be due to the loss of electrostatic interactions with the tsDNA backbone rather than changes in steric bulk. We obtained a separate MFEP for each of the mutations (Figure 4A,B).

For the R856L mutant, the average free energy of the IS increased significantly to 8.45 kcal/mol compared to 3.69 kcal/mol for the wildtype. The Fin. state energy difference between R856L and the wildtype, however, remained relatively similar (WT: 3.56 kcal/mol, R856L: 4.47 kcal/mol). Since residue 856 is distal to the cleavage site, its impact on the stability of the Fin. state is minimal. The major influence of this mutation was observed during the early stages of the MFEP.

In contrast, the R904L mutation resulted in an even more pronounced increase in the average free energy of the IS, rising to 9.02 kcal/mol, also from a baseline of 3.69 kcal/mol in the wildtype. The difference in the Fin. state energy between R904L and the wildtype was considerably larger (R904L: 8.98 kcal/mol). Given the proximity of residue 904 to the cleavage site, this mutation disrupted the stability of the cleavage complex, thereby elevating the entire MFEP.

### 2.4. Cleavage Activity Depends on Glu662^RuvC^

**Figure 5:**
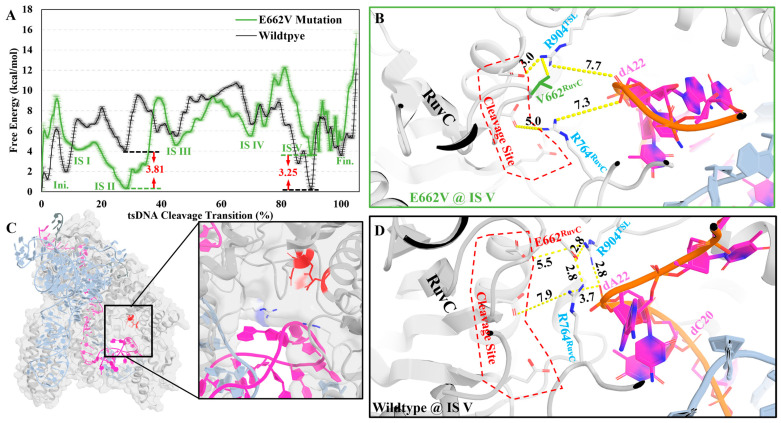
Comparison between the wildtype and mutation structure around residue 662^RuvC^. (**A**) The comparison between the wildtype and the E662V mutation MFEP; wildtype in black and E662V colored in green; red number indicates the difference between the same IS for different MFEPs. (**B**) The IS V for the E662V mutation protein is colored in white, and sgRNA is colored in pale blue; tsDNA is colored in magenta, and Val662^RuvC^ is colored in green. (**C**) The spatial location of the structure analysis; protein is colored in white cartoon or stick mode, sgRNA is colored in pale blue in cartoon mode; tsDNA is colored in magenta as a cartoon model. (**D**) The IS V for wildtype, protein is colored in white and sgRNA are colored in pale blue; tsDNA is colored in magenta.

At the terminus where Arg904^TSL^ and Arg764^RuvC^ anchor the tsDNA, a delicate balance is maintained by the presence of Glu662^RuvC^, strategically positioned at the edge of a β-sheet and complemented by the highly negatively charged cleavage site. This configuration is imperative for the seamless integration of the tsDNA into the cleavage site, facilitating the critical final step for DNA cleavage (Figure 5D). Thus, not only do Arg904^TSL^ and Arg764^RuvC^ secure one end of the cleavage target, but they also operate under the directive influence of Glu662^RuvC^ and the cleavage site, ensuring a smooth transition of the tsDNA into the cleavage site.

To validate the proposed reason for the stability of the IS V hypothesis, we sought to disrupt the delicate balance within this state and thereby confirm its significance in the transition mechanism. As previously highlighted, the orientation of positive residues is governed by negative ones, a principle exemplified in IS V through the regulatory influence of Glu662^RuvC^ and the cleavage site over Arg764^RuvC^ and Arg904^TSL^ (Figure 5D).

To test this hypothesis, a mutation study was designed, focusing on Glu662^RuvC^ to ensure that any observed effects were indeed due to its electrostatic interaction with the two arginines (Arg764^RuvC^ and Arg904^TSL^) rather than simply its steric bulk. Glu662^RuvC^ was substituted with valine (E662V) to control for steric effects, given that glutamic acid and valine have comparable volumes (glutamic acid: 138.4 Å^3^, valine: 140.0 Å^3^).

A new and optimized path was obtained upon introducing this mutation; therefore, a new MFEP was obtained for the E662V mutant (Figure S6C). Analyzing the MFEP revealed a striking alteration compared to the wildtype: the stable IS V was notably absent (Figure 5A). Delving into the structural details illuminated the consequences of this modification. Without Glu662^RuvC^’s regulatory influence, Arg764^RuvC^ and Arg904^TSL^ shifted their positions, getting closer to the highly negatively charged cleavage site (Asp659^RuvC^, Glu756^RuvC^, and Asp922^RuvC^) rather than maintaining their stabilizing interactions with the negatively charged tsDNA backbones (Figure 5B). This misalignment led to increased mobility of the tsDNA, resulting in an elevation in the free energy (3.25 kcal/mol) of what would have been IS V.

Furthermore, the absence of Glu662^RuvC^’s control allowed Arg764^RuvC^ to linger within the cleavage site for extended durations, inadvertently fostering an unseen stable IS II (free energy decreased by 3.81 kcal/mol), trapping the protein in this configuration instead of the Fin. state, and thus further reducing the overall cleavage efficiency. This unintended consequence underscores the profound impact of Glu662^RuvC^ on maintaining the progression and integrity of intermediate states, highlighting its pivotal role in the intricate choreography of the cleavage process (Figure S8).

## 3. Discussion

Previous studies have provided extensive static structural information on each stage of the cleavage process and the biochemical properties of various domains within *Plm*Cas12e [8,17,18,19,21]. This enzyme can be divided into seven distinct domains, with TSL and RuvC identified as particularly crucial for the transition from the ntsDNA-cleaved to the tsDNA-loaded state. The NTSB domain is essential for initiating the sgRNA-tsDNA double helix; even the structure of the sgRNA has been confirmed to play a significant role in cleavage efficiency.

Our TAPS approach offers insight into the dynamic process by which tsDNA is transported to the cleavage site. Initially, the movements of Helical-I, Helical-II, and BH domains constrain and deform the sgRN–-tsDNA double helix. During this phase, two positively charged residues, Arg768^RuvC^ and Arg856^TSL^, control the backbone of the tsDNA, preventing its backward movement. Subsequently, the TSL domain captures the sgRNA, further limiting the tsDNA’s access to the cleavage site, effectively restricting its proximity. Finally, the positively charged residues (Arg768^RuvC^, Lys898^TSL^, and Arg904^TSL^) straighten the tsDNA’s backbone, allowing the bases to flip out of the cleavage site, thereby facilitating the cleavage. In the final stage, we found that Glu662^RuvC^ is a key residue coordinating with Arg764^RuvC^, preventing it from occupying the cleavage site prematurely until the tsDNA is properly positioned. A mutation of Glu662^RuvC^ to valine results in a stable intermediate state where Arg764^RuvC^ occupies the cleavage site too early, inhibiting the efficient loading of tsDNA. This finding underscores the importance of the interaction between Glu662^RuvC^ and Arg764^RuvC^ in maintaining the correct conformation necessary for successful cleavage.

Compared to other mutations discussed in this article, the wildtype exhibits the lowest delta (energy difference between the Ini. and Fin. states) and total forward free energy barrier. Residues near the cleavage site, such as Glu662^RuvC^ and Arg904^TSL^, have a more substantial impact on the stability of the cleavage site, increasing the free energy by 1.52 kcal/mol and 5.42 kcal/mol, respectively (Figure 4D, Table 1). In contrast, residue 856, which is distal to the cleavage site, primarily stabilizes the tsDNA during the transition, having a lesser effect on the cleavage site’s stability. With another residue (Arg768^RuvC^) also aiding in tsDNA stabilization, the overall impact of residue 856 on the total forward free energy barrier is minimal, with an increase of only 2.36 kcal/mol. These observations suggest that the transition from ntsDNA to tsDNA involves two main components: the stability of the cleavage site and the forward energy barriers. Positively charged residues like Arg856^TSL^, Arg768^RuvC^, and Lys898^TSL^ stabilize the tsDNA, ensuring an efficient transition, while polar residues such as Glu662^RuvC^, Arg764^RuvC^, and Arg904^TSL^ are critical for maintaining a stable cleavage site.

Our findings extend previous static structural data by providing a detailed mechanistic understanding of how residues and domains coordinate to facilitate the precise loading of tsDNA. This knowledge is applicable to the development of advanced gene-editing technologies. By leveraging the new insights into the interactions between Glu662^RuvC^ and Arg764^RuvC^, researchers can design Cas12e variants that optimize the stability of the final state, making the transition more favorable. However, it’s crucial to acknowledge the limitations of our computational approach. Experimental validation is necessary to confirm the functional significance of the identified residues. Additionally, exploring the TSL domain’s interaction with sgRNA and its influence on tsDNA mobility could provide a more comprehensive understanding of the entire loading process.

Future research should validate computational predictions through in vivo or in vitro experiments and examine the impact of modifying these residues on Cas12e’s functionality. Additionally, a deeper investigation into the TSL domain’s role in confining tsDNA through its interaction with sgRNA could enhance our understanding and contribute to the development of more efficient Cas12e-based gene-editing systems.

## 4. Methods

### 4.1. Homology Modeling

The cryo-EM structures of the non-target strand cleavage state (PDB ID: 7WAY) and target strand cleavage state (PDB ID: 7WAZ) were obtained from the RCSB Protein Data Bank (rcsb.org) for use in modeling and simulations [8]. Some residues in the flexible loop regions were missing from these structures. To address this, the 7WAY structure was imported into ChimeraX [45]. The 7WAY protein sequence was subjected to a BLAST [46] search to identify the best match for homology modeling. The top matching sequence was then uploaded to the MODELLER server [47], which generated five potential structures. The structure with the lowest Discrete Optimized Protein Energy (DOPE) score was selected. This structure was aligned with 7WAY, and the missing protein segments were added accordingly.

### 4.2. System Preparation

For all simulations, we prepared the input files using GROMACS-2022.3 [48]. The homology-modeled structure was placed in a solvated box of dimensions 11.800 × 17.716 × 15.814 nm using the TIP3P [49] water model. To achieve neutralization and match the conditions of the cleavage assays, 0.4 M K^+^/Cl^−^ and 0.05 M Mg^2+^/Cl^−^ ions were added. The Amber14SB [50] force field was applied to all standard amino acids, nucleic acids, and ions.

### 4.3. Molecular Dynamics Simulations

Molecular dynamics (MD) simulations were conducted to relax and evaluate the stability of the modeled structures. These simulations were executed with GROMACS-2019.4, employing the Amber14SB force field for system interactions. Energy minimization was achieved over 5000 steps using the steepest descent algorithm, with backbone and side-chain restraints of 400 kJ/mol and 40 kJ/mol, respectively. This was followed by a 125 ps NVT simulation at 310 K for solvent equilibration and temperature coupling, using a Nose-Hoover thermostat and maintaining the same restraints. The production MD simulations, spanning 40 ns with a 2-fs time step, employed a Parrinello–Rahman barostat and a Nose–Hoover thermostat without restraints on backbones or side chains. Long-range electrostatic interactions were calculated using the Particle-Mesh Ewald (PME) method, with short-range electrostatic and van der Waals interactions both set to a 9 Å cutoff. Hydrogen bonds were constrained using the LINCS algorithm.

### 4.4. Generation of Initial Path

Targeted MD (tMD) [51,52] simulations were performed with GROMACS-2019.4 and PLUMED-2.5.3 [53,54], transitioning the complex from the non-target strand to the target strand cleavage state. The non-target strand and target strand cleavage state structures underwent relaxation through a 40-ns MD simulation. In tMD, an additional harmonic bias potential was applied, described by $V_{spring}(S,t)=k(t){\left[S\left(t\right)-S_{T}\right]}^{2}/2$, where $V_{spring}(S,t)$ is the harmonic potential on the collective variable, $k\left(t\right)\;$ is the spring force constant, S(t) is the current structure, and $S_{T}$ is the target structure. The spring constant k(t) was set to different values at specified times: 0, 10,000, 100,000, 200,000, 500,000, 1,000,000, 2,000,000, and 2,500,000 kJ/(mol∙nm^2^) at 0.2, 0.8, 1, 2, 3, 4, and 5 ns, respectively. The bias was applied to all key atoms of the *Plm*Cas12e complex. At the same time, structural alignments were conducted using the protein’s Cα atoms and the nucleic acid’s phosphorus atoms within the *Plm*Cas12e complex. All targeted MD’s parameters are in the following table (Table 2).

### 4.5. Path Optimization

Optimizing the path is crucial for determining the minimum free energy paths (MFEPs), which are more physically accurate than the initial trajectories. We utilized the TAPS method for this optimization, using an in-house Python script incorporating GROMACS, PLUMED, and Concorde.

The traveling-salesman-based automated path-searching (TAPS) method was applied to the initial pathway generated from a targeted simulation to obtain the minimum free energy path. This approach provides deeper insights into the free energy landscape and its relationship with the molecular structure. In this method, the path-collective-variable (PCV) technique is implemented, which defines the free energy landscape using two components: PCV-s and PCV-z; PCV-z is a measure that quantifies how different the current state of an MD simulation is from a set of reference states. It is calculated using a metric that assesses the distance between configuration states, along with a smoothing parameter λ. The value of PCV-z ranges from 1 to N, where N represents the number of reference states. Typically, PCV-z is normalized to a range of 0 to 1, with 0 indicating that the system is exactly at one of the reference states and 1 indicating that it is as far away from all reference states as possible. PCV-z can be used to monitor the progress of a simulation or to identify conditions that deviate significantly from the reference states. PCV-s, on the other hand, can be employed with well-tempered metadynamics to enhance sampling and facilitate non-local exploration of the free energy surface. This allows for the discovery of optimal (low free energy) paths that may be distant from the initial guess path and are not easily found using other path-based methods. After sampling each node, the centroids of conformations with the median value of PCV-z are selected as the nodes for the new path. The order of these nodes is then optimized using the traveling-salesman algorithm. Finally, reparameterization, completed by targeted MD, inserts additional nodes between pairs that are farther apart than a specified threshold, ensuring the resolution of the new path is maintained. This iterative process continues until a converged path is achieved. For further details, please refer to Zhu et al. (2019) [42].

The simulations were performed at 310 K in the NVT ensemble, controlled by a velocity-rescale thermostat. Conformations were selected from the tMD trajectory at 1.0 Å intervals to create the initial path. The RMSD between conformations was calculated using all key atoms of the *Plm*Cas12e complex, while structural alignments employed Cα atoms for proteins and phosphorus atoms for nucleic acids. Each TAPS iteration involved 4000 ps of sampling, with Gaussians of 2 kJ/mol height and 1.0 width deposited every 5 ps, and frames were recorded at the same interval. Convergence after optimization was evaluated using multidimensional scaling (MDS) and PCV-z analysis [55]. The parameters used in TAPS optimization is in the following table (Table 3).

### 4.6. Free Energy Calculation

We utilized umbrella sampling for free energy calculations to assess the likelihood of tsDNA cleavage transitions and identify transition and intermediate states. These calculations were executed with GROMACS-2019.4 and PLUMED-2.5.3. We derived free energy profiles of the MEFP along PCV-s, which indicate progression along the MEFP. Sampling windows were restricted within 0.04 nm of the MEFP by applying a harmonic wall potential with a force constant of 2,000,000 kJ/(mol∙nm^2^) at PCV-z = 0.0064 nm^2^. The RMSD between conformations was measured using all key atoms of the *Plm*Cas12e complex, while structural alignments were based on the Cα atoms for proteins and phosphorus atoms for nucleic acids within the *Plm*Cas12e complex. We chose a window size of 0.25 nm along the MEFP, with each window using a force constant of 200 kJ/(mol∙nm^2^). Each window underwent simulations for a minimum of 4 ns (Figure S9). The weighted histogram analysis method (WHAM) was employed to generate the complete free energy profile [56,57]. Parameters for umbrella sampling are detailed in the following two tables (Table 4 and Table 5).

## 5. Conclusions

Our study provides significant insights into the intricate mechanisms underlying tsDNA loading by *Plm*Cas12e. We elucidated the importance of several positively charged residues (Arg856^TSL^, Arg768^RuvC^, Lys898^TSL^, Arg904^TSL^, and Arg764^RuvC^) that guide the tsDNA backbone toward the cleavage site. Of particular note is the key gatekeeper role played by the negatively charged Glu662^RuvC^ in coordinating Arg764^RuvC^, preventing it from occupying the cleavage site and thus maintaining a delicate balance that ensures smooth tsDNA loading into the cleavage site. Additionally, our study highlighted the involvement of the TSL domain and its interaction with the sgRNA in confining the mobility of the tsDNA, optimizing its backbone orientation for cleavage. The rearrangement of charged residues, especially Arg764^RuvC^, Arg904^TSL^, and Lys955^RuvC^, ensured that tsDNA could only adopt a specific orientation that facilitated its cleavage. These arrangements were influenced by the strategic positioning of charged residues, and these arrangements emphasize the importance of electrostatic interactions within the Cas12e protein, which offered a foundation for the rational engineering of Cas12e for enhanced specificity and efficiency.

## Figures and Tables

**Table 1:** Details of the energy delta between the Ini. and Fin. states and forward energy barriers for wildtype, E662V, R856L, and R904L.

System	Δ (kcal/mol) (Stability)	Total FWD Barrier (kcal/mol) (Efficiency)
Wildtype	3.56	40.29
Glu662Val	5.08	51.84
Arg856Leu	4.47	42.67
Arg904Leu	8.98	40.76

**Table 2:** Details of the targeted MD for *Plm*Cas12e and *Plm*Cas12e, E662V, R856L, and R904L.

System		*Plm*Cas12e (WT)	*Plm*Cas12e (E662V)	*Plm*Cas12e (R856L)	*Plm*Cas12e (R904L)
Complex Size		22,203 atoms	22,204 atoms	22,199 atoms	22,199 atoms
K^+^/Mg^2+^/Cl^−^		940/100/996	939/100/996	941/100/996	941/100/996
Water		96,136	96,183	96,173	96,168
Temperature		310 K			
Target State		TS Cleavage State			
Atom set	Structural Alignment	Amino Acid: Cα, Nucleic Acid: P			
	RMSD Calculation	All Heavy Atoms			
Force Constant		Multiple			
Frame Record Frequency		2 ps			
Total Sampling Time		5500 ps			

**Table 3:** The parameters used in TAPS optimization for the systems in the present work.

System		*Plm*Cas12e (WT)	*Plm*Cas12e (E662V)	*Plm*Cas12e (R856L)	*Plm*Cas12e (R904L)
Tolerant Distance for Neighboring Nodes		2 Å	1.85 Å	1.65 Å	1.7 Å
Sampling Time Each Iteration		4000 ps			
Temperature		310 K			
Atom set	Structural Alignment	All residues within 10 Å of the R-loop and gRNA 1–3 & 26–57 during the initial tMD path, and the nucleic acid themselves.			
	RMSD Calculation				
Well-Tempered Metadynamics Simulation	Gaussian Height	2			
	Gaussian Width	1			
	Bias Factor	10			
	Length of tMD	10 ps			
Force Constant of tMD		150,000 kJ/mol·nm^2^			

**Table 4:** Details of umbrella sampling for the systems in the present work.

System		*Plm*Cas12e (WT)	*Plm*Cas12e (E662V)	*Plm*Cas12e (R856L)	*Plm*Cas12e (R904L)
Node Number in Final Path		64	58	66	55
Insert Gap		0.25 PCV-s			
Atom set	Structural Alignment	All residues within 10 Å of the R-loop and gRNA 1–3 & 26–57 during the initial tMD path, and the nucleic acid themselves.			
	RMSD Calculation				
Total Number of Nodes for Umbrella Sampling		256 + 12	242 + 13	264 + 2	220 + 7
PCV-based Umbrella Sampling	Position for z-Wall Potential	0.0064 nm^2^			
	Force Constant for PCV-z	20,000,000 kJ/mol·nm^2^			
	Force Constant for PCV-s	200 kJ/ mol·nm^2^			
	Sampling Time	4 ns			

**Table 5:** Details of atom set selection for each individual amino acid and nucleic acid.

Amino/Nucleic Acid	Align Atom	RMS Atom
Ala	Cα	/
Gly	Cα	/
Ile	Cα	CB, CG1
Leu	Cα	CG
Pro	Cα	/
Val	Cα	CB
Phe	Cα	CG
Trp	Cα	CG, NE1
Tyr	Cα	CG, CZ
Asp	Cα	CG
Glu	Cα	CD
Arg	Cα	CZ
His	Cα	CG
Lys	Cα	CE
Ser	Cα	CB
Thr	Cα	CB
Cys	Cα	CB
Met	Cα	CG
Asn	Cα	CG
Gln	Cα	CD
dA	P	N9, C2, C6
dC	P	N1, N3, C5
dG	P	N9, C2, C6
dT	P	N1, N3, C7
A	P	N9, C2, C6
C	P	N1, N3, C5
G	P	N9, C2, C6
U	P	N1, N3, C5

## Data Availability

The data have not been uploaded to a publicly available repository, and the data that support the findings of this study are available upon reasonable request to the corresponding author, Zhu.

## Supplementary Materials

The following supporting information can be downloaded at: https://www.mdpi.com/article/10.3390/molecules29215036/s1, Figure S1: Comparison between DpbCas12e and PlmCas12e in ntsDNA cleavage state; Figure S2: Before and after TAPS optimization; Figure S3: Structures in detail; Figure S4: Statistical analysis along the tsDNA cleavage transition; Figure S5: Area the tsDNA can access; Figure S6: The E662V mutation convergence and the MFEP; Figure S7: The flexible, positively charged residue is controlled by negatively charged or polar residues based on or around a rigid structure; Figure S8: Compare between the wild-type and E662V mutation for IS II; and Figure S9: Sample distribution in all windows of the umbrella sampling along the PCV-s for wildtype and E662V mutation.
