# Supplementary material for: PlmCas12e Utilizes Glu662 to Prevent Cleavage Site Occupation by Positively Charged Residues Before Target Strand Cleavage
Source: Molecules. 2024 Oct 25;29(21):5036. doi: 10.3390/molecules29215036 (PMC11547573; doi:10.3390/molecules29215036)
Supplement: Supplementary file 1 [file molecules-29-05036-s001.zip › molecules-3193966-supplementary.pdf]

PlmCas12e Utilizes Glu662 to Prevent Cleavage Site Occupation by Positively Charged Residues Before Target Strand Cleavage

Jinchu Liu[1], and Lizhe Zhu[1,*]

[1]*Warshel Institute for Computational Biology, School of Medicine, The Chinese University of Hong Kong - Shenzhen, Shenzhen 518172, China*

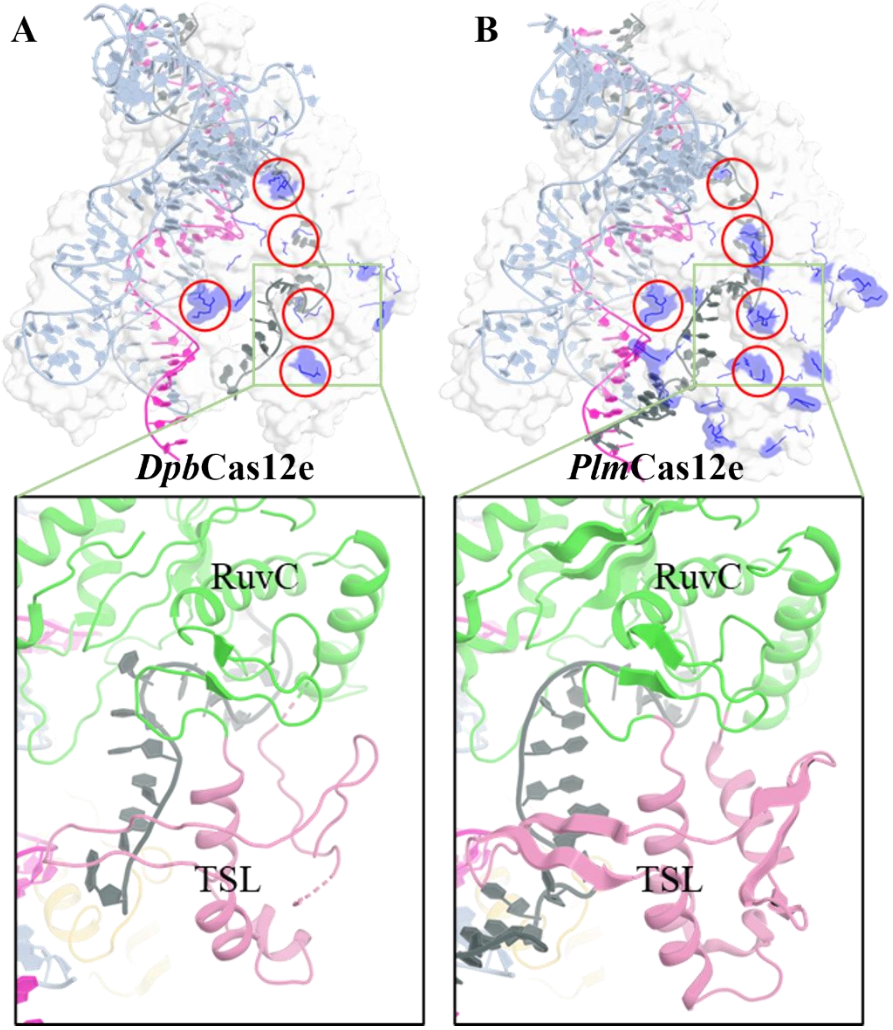

Figure S1. Comparison between *Dpb*Cas12e and *Plm*Cas12e in ntsDNA cleavage state. Red circles are the positively charged residues that are identical in position for both Cas12e system. A. *Dpb*Cas12e at ntsDNA cleavage state, sgRNA is colored in pale blue, tsDNA is color in magenta, ntsDNA is colored in grey, positively charged residues are color blue in stick mode; RuvC domain colored in green and TSL domain colored in pink. B. *Plm*Cas12e at ntsDNA cleavage state, sgRNA is colored in pale blue, tsDNA is color in magenta, ntsDNA is colored in grey, positively charged residues are color blue in stick mode; RuvC domain colored in green and TSL domain colored in pink.

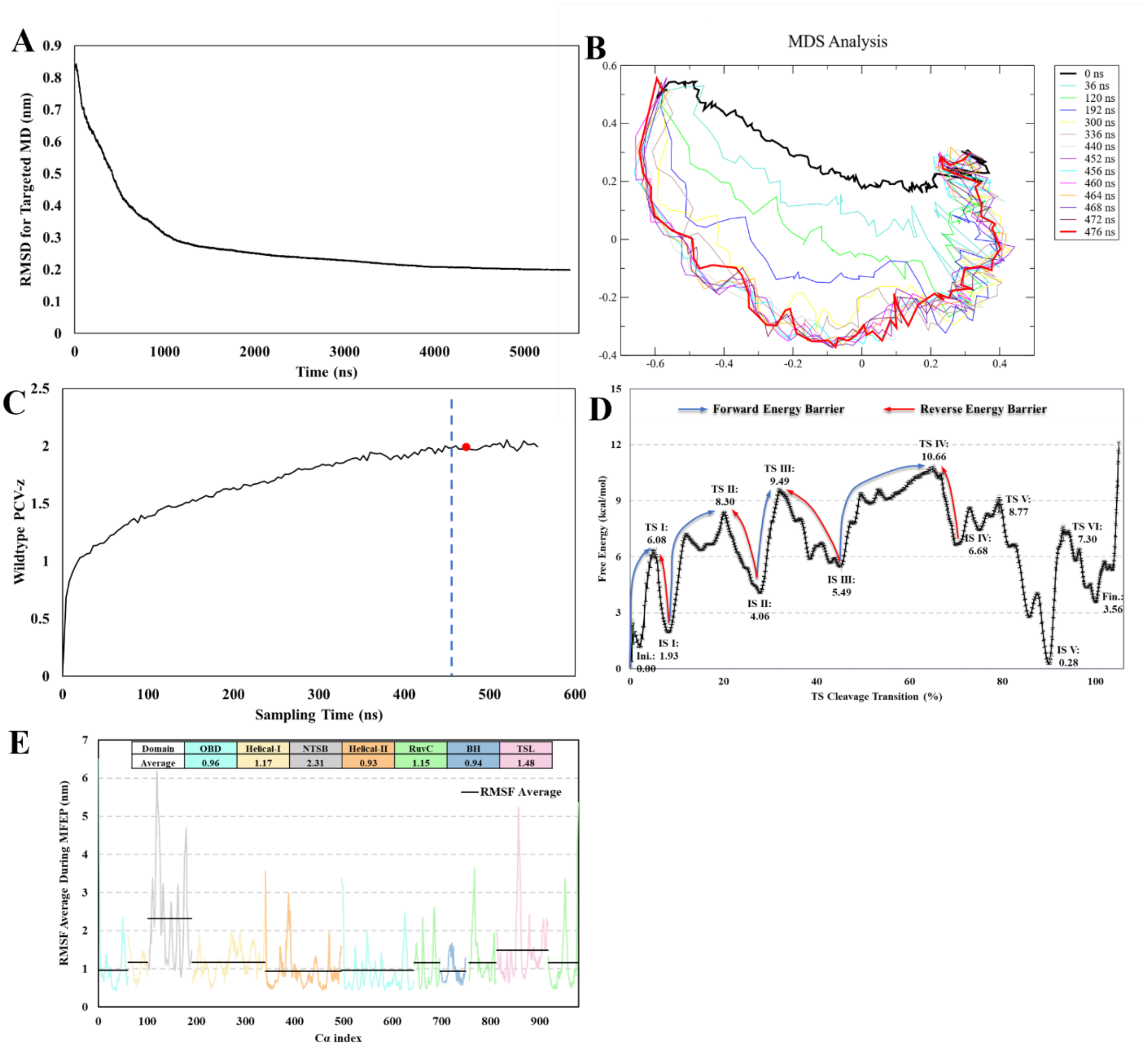

Figure S2. Before and after TAPS optimization. A. The RMSD calculation for the Targeted MD uses PDB: 7WAZ as the target structure. B. *Plm*Cas12e protein, gRNA, TS, and NTS complex are colored in gray, atoms for alignment are colored in cyan, and atoms for RMSD calculation are colored in wheat. C. For the PCV-z value with respect to sampling time, the blue line indicates where the convergence has been reached, and the red dot is the specific trajectory we pick out for MFEP calculation. D. The Multidimensional Scaling (MDS) analysis was performed on different trajectories at each sampling time to check convergence; the bold black line is the initial trajectory, and the bold red line is the trajectory for MFEP calculation. E. the tsDNA cleavage transition MFEP in detail, blue arrows represent the forward barriers and red arrows represents backwards barriers. F. Average RMSF for all residues' Cα. OBD domain colored in cyan, Helical I domain colored in yellow, NTSB domain colored in grey, Helical II domain colored in orange, RuvC domain colored in green, BH domain colored in blue, and TSL domain colored in pink.

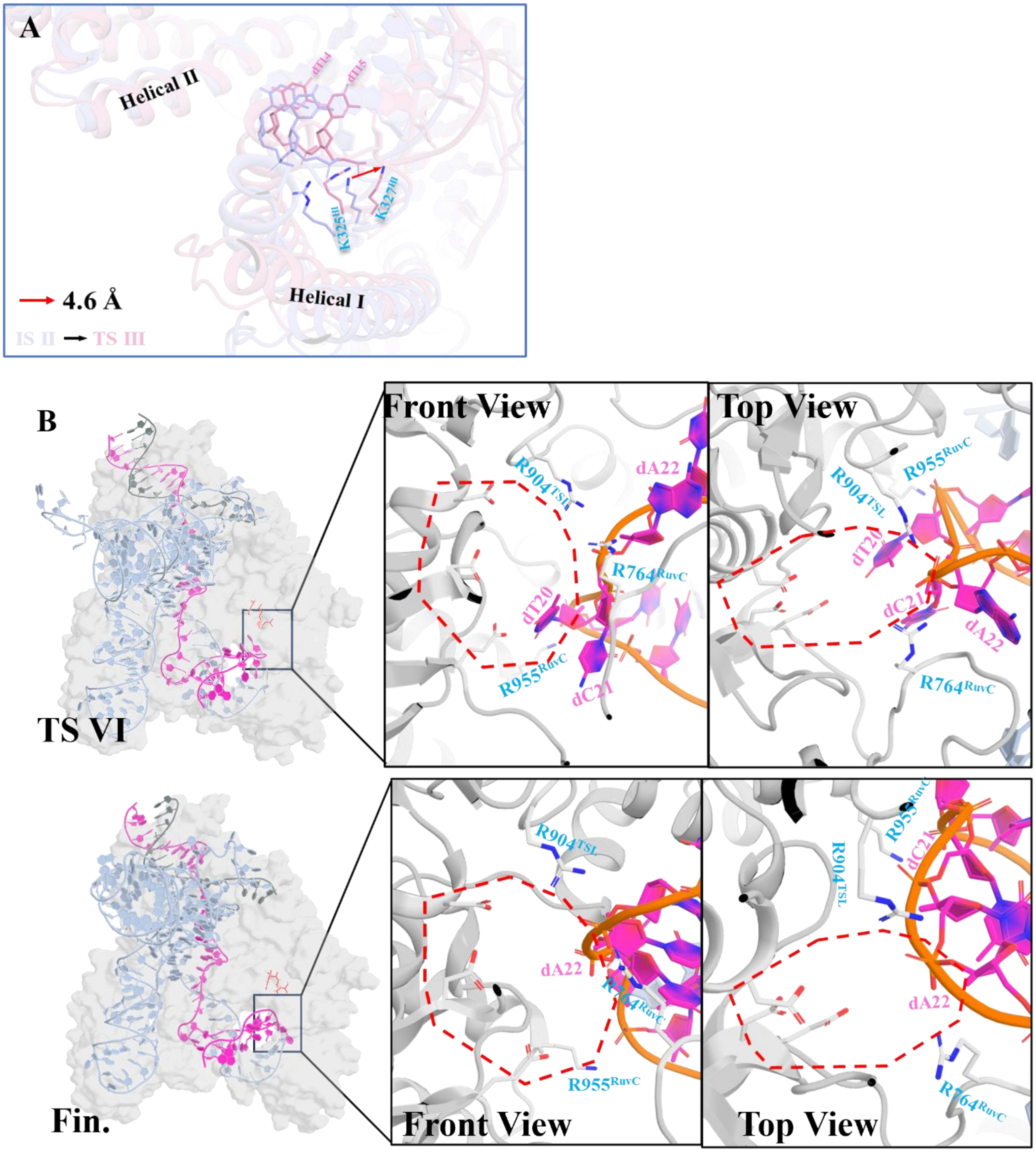

Figure S3. Structures in detail. A. the structure changes from IS II to TS III for wildtype *Plm*Cas12e, the red arrow indicates the movement of residues and nucleotide, and the number indicate displacement in space. B. Shows the wildtype *Plm*Cas12e at TS VI and Fin. states which is the state right before cleavage, the three positively charged residues are colored in blue, and the backbone of the tsDNA is colored in orange and magenta, the cleavage site is indicated by the red dashed circle.

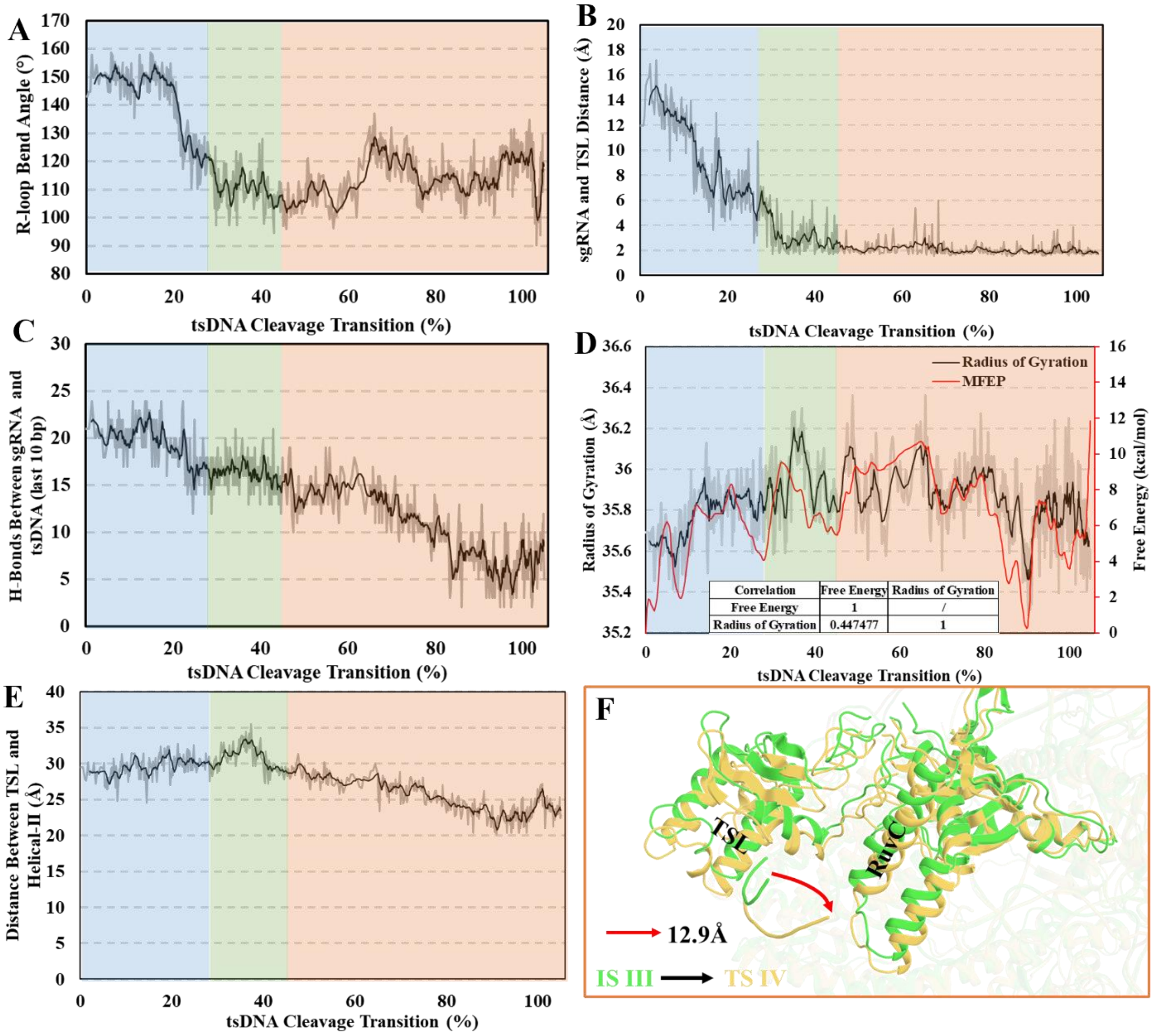

Figure S4. Statistical analysis along the tsDNA cleavage transition. Stage 1 is from Ini. to IS II, colored in blue, Stage 2 is from IS II to IS III colored in green, Stage 3 is from IS III to Fin., colored in orange. A. The bending angle of the R-loop, blue background is the first stage, green background is the second stage, and orange color is the third stage. B. The distance between the sgRNA and the TSL domain, blue background is the first stage, green background is the second stage, and orange color is the third stage. C. The hydrogen bond number between the last 10 bp of the R-loop, blue background is the first stage, green background is the second stage, and orange color is the third stage. D. The radius of gyration or the compactness of protein, red line is the MFEP overlay on top, and the table is showing the correlation between the MFEP and the radius of gyration, blue background is the first stage, green background is the second stage, and orange color is the third stage. E. The distance between the Helical II and the TSL domain, blue background is the first stage, green background is the second stage, and orange color is the third stage. F. the structure changes from IS III to TS IV for wildtype PlmCas12e, the red arrow indicates the movement of residues and nucleotide, and the number indicate displacement in space.

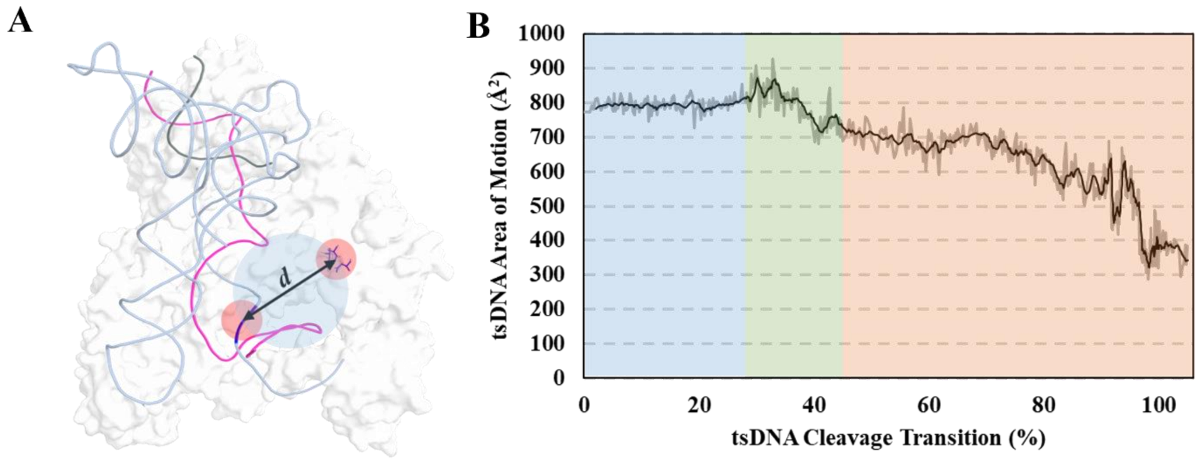

Figure S5. Area the tsDNA can access. Stage 1 is from Ini. to IS II, colored in blue, Stage 2 is from IS II to IS III colored in green, Stage 3 is from IS III to Fin., colored in orange. A. Red circle indicates the position or the cleavage site and the G12 and A13 of the sgRNA, the arrow indicates the distance between this two groups center of mass, using this distance as a diameter to calculate the accessible area for the tsDNA. B. tsDNA Area of motion or the accessible area by the tsDNA, blue background is the first stage, green background is the second stage, and orange color is the third stage.

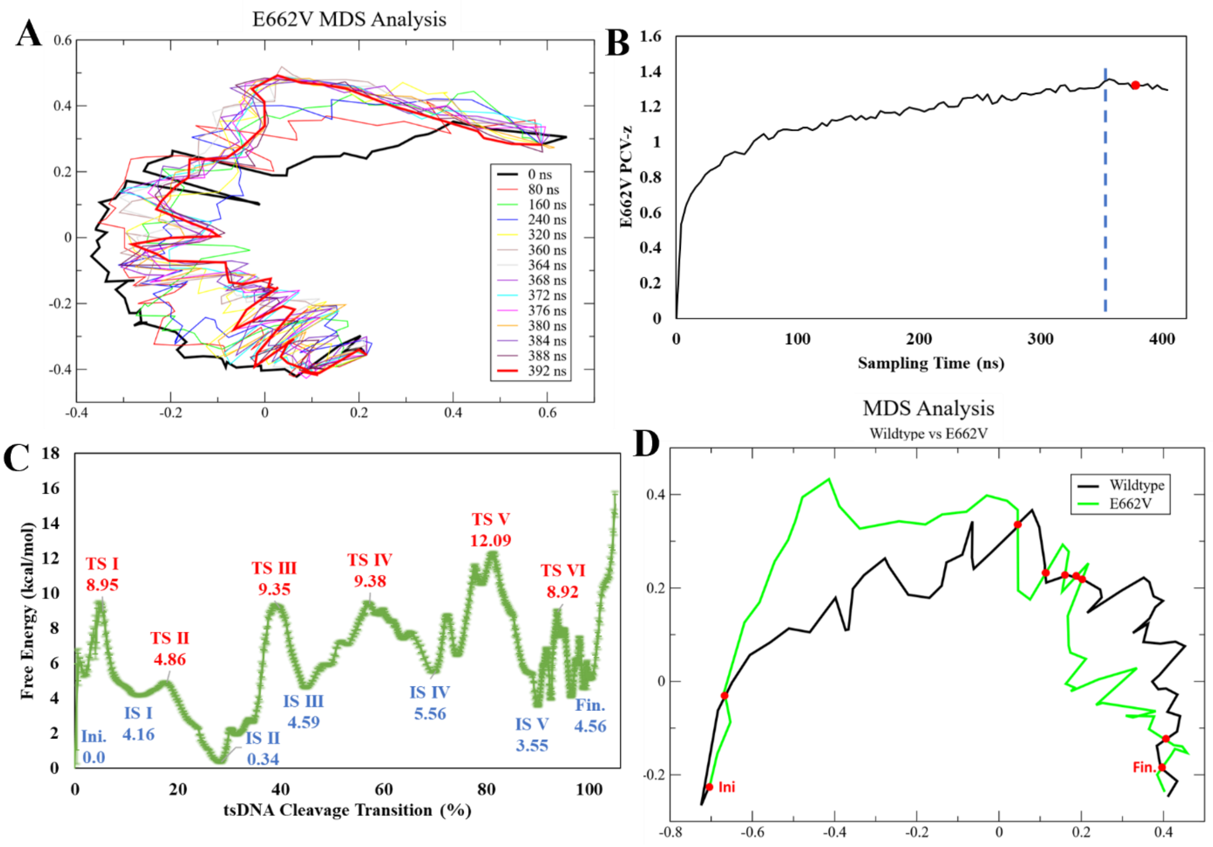

Figure S6. The E662V mutation convergence and the MFEP. A. The Multidimensional Scaling (MDS) analysis was performed on different trajectories at each sampling time to check convergence; the bold black line is the initial trajectory, and the bold red line is the trajectory for MFEP calculation. B. For the PCV-z value with respect to sampling time, the blue line indicates where the convergence has been reached, and the red dot is the specific trajectory we pick out for MFEP calculation. C. The E662V mutation MFEP for the NTS to TS cleavage transition. Free energy for transition states is highlighted in red, and intermediate states are in blue; errors are shown in the error bars. D. The MDS analysis for the wild-type and the E662V mutated trajectory, the black line is the wild-type, and the green line is the mutated; the red dots are where the two trajectories crossed over, those specific structures that are used for trajectory alignment.

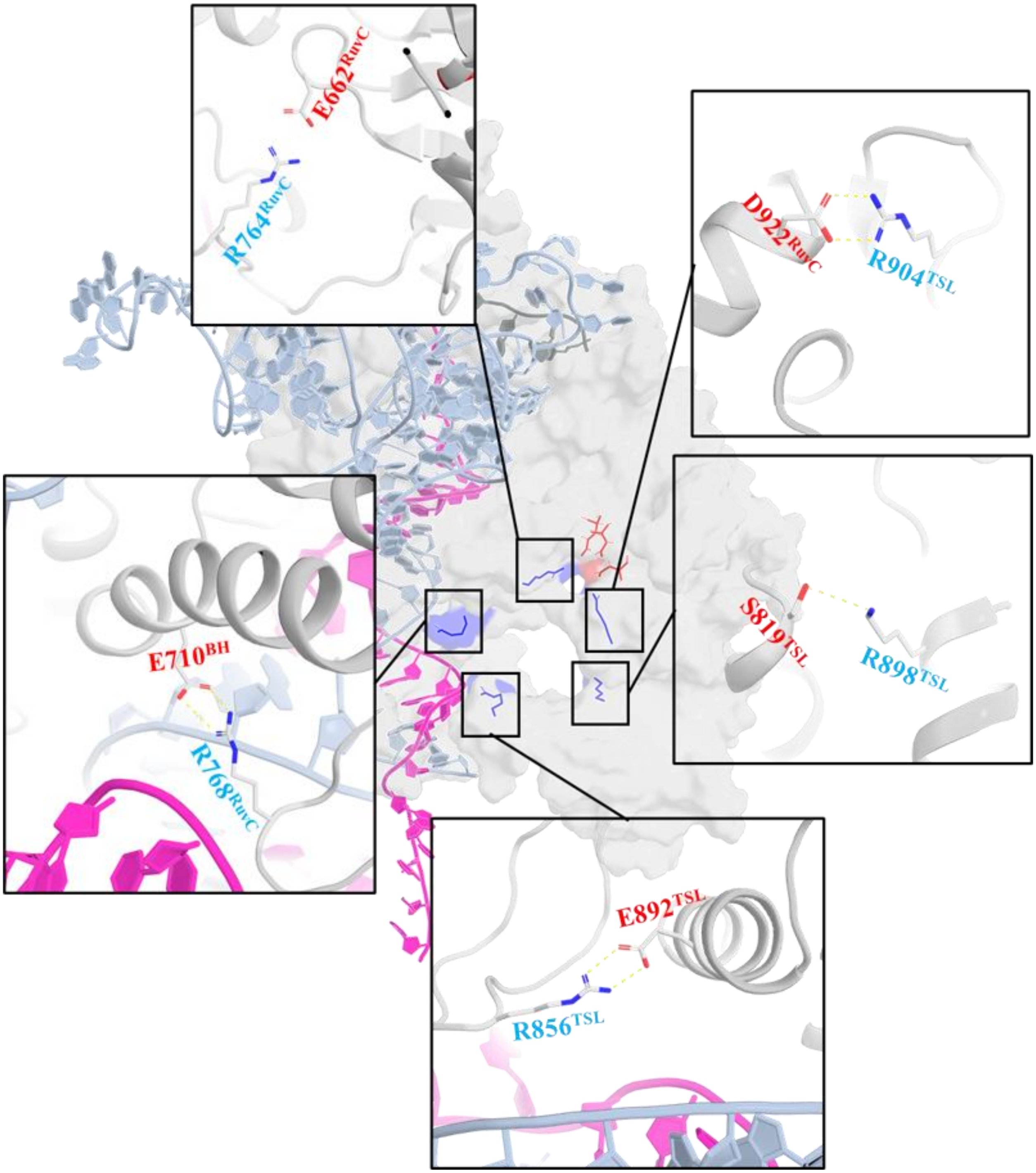

Figure S7. The flexible, positively charged residue is controlled by negatively charged or polar residues based on or around a rigid structure. It shows the five positively charged residues involved in the tsDNA's translocation. Blue labels are positively charged residues, and red labels are negatively charged residues. The background is the initial structure of the trajectory, and the enlarged portion is at different times along the trajectory.

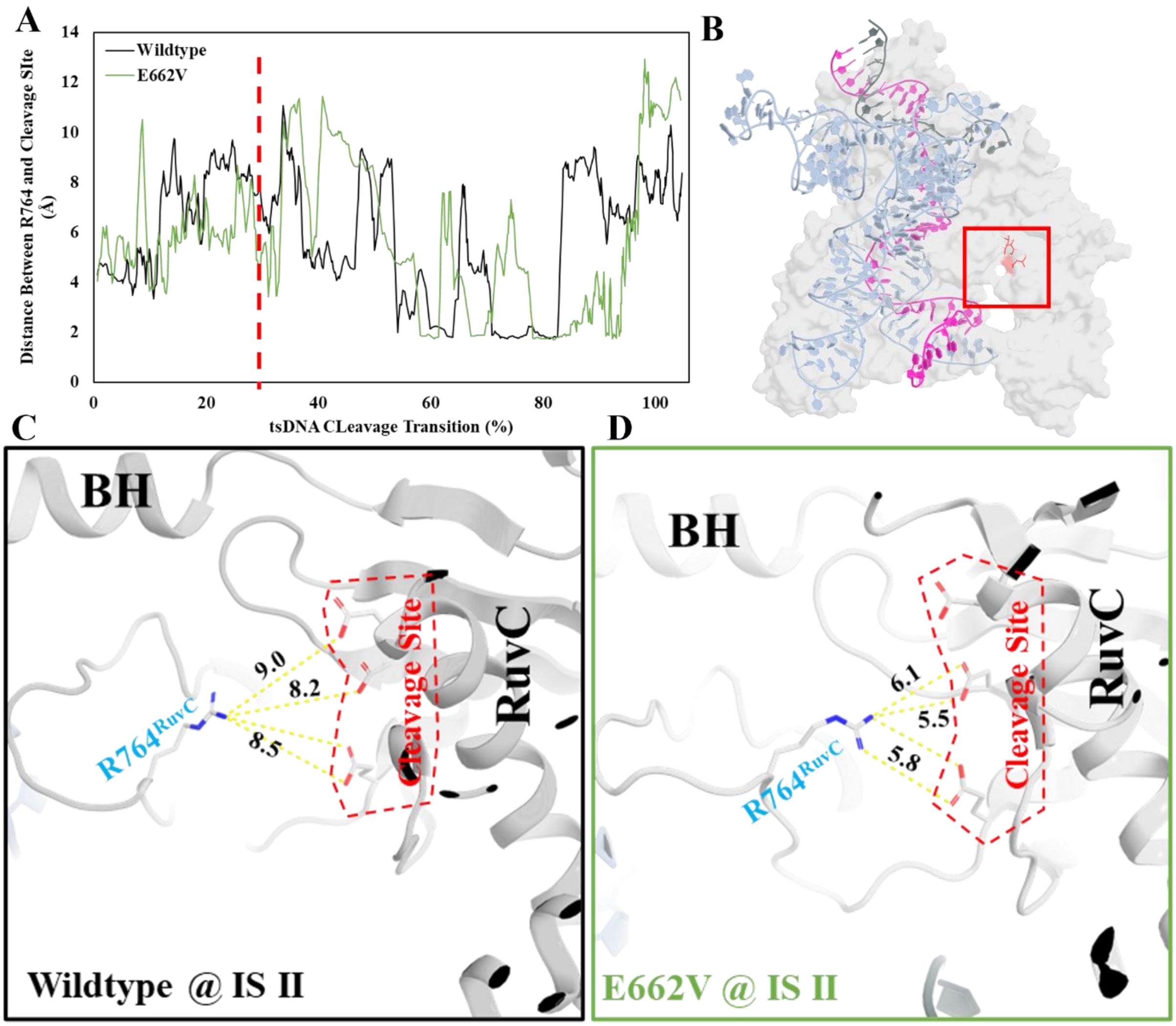

Figure S8. Compare between the wild-type and E662V mutation for IS II. A. The distance between the positively charged Arg764^{RuvC} and the negatively charged cleavage site; the black line is wild-type, and the green line is the mutant; the red dashed line indicates the IS II. B. the IS II's conformation, protein, and sgRNA is colored in pale blue, and tsDNA is colored in magenta, the red square indicates the spatial location for C and D. C, D. The specific distance difference between IS II for wild-type and mutant between Arg764^{RuvC} and the cleavage site.

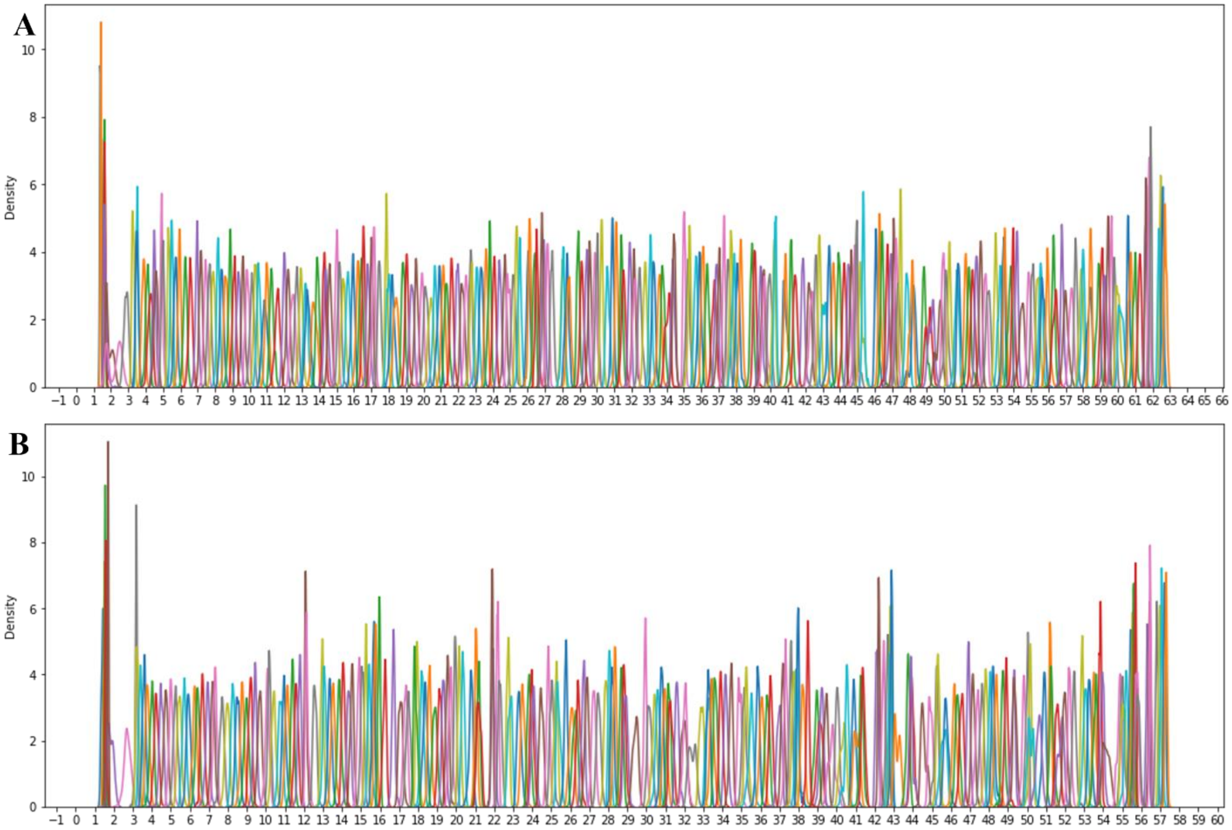

Figure S9. Sample distribution in all windows of the umbrella sampling along the PCV-s for wildtype (A) and E662V mutation (B).
